# *S. aureus* infections in hospitalized children with hematological, hepatological or kidney diseases over a 16-year period at a tertiary care hospital in Sweden: a retrospective observational study

**DOI:** 10.1186/s13052-026-02289-4

**Published:** 2026-06-04

**Authors:** Susan Farmand, Hannah Kurz, Jack Isaksson, Milan Chromek, Birgitta Henriques-Normark, Mikael Sundin

**Affiliations:** 1 Section of Hematology, Immunology and HSCT, Astrid Lindgren Children’s Hospital, Karolinska University Hospital, Stockholm, Sweden; 2 Department of Microbiology, Tumor and Cell Biology, Karolinska Institutet, Stockholm, Sweden; 3 Division of Pediatric Stem Cell Transplantation and Immunology, University Medical Center Hamburg-Eppendorf, Hamburg, Germany; 4 Department of Pediatrics, University Medical Center Hamburg-Eppendorf, Hamburg, Germany; 5 Pediatric Nephrology Unit, Karolinska University Hospital Huddinge, Stockholm, Sweden; 6 Division of Pediatrics, Department of Clinical Science, Intervention and Technology, Karolinska Institutet, Stockholm, Sweden; 7 Department of Clinical Microbiology, Karolinska University Hospital, Solna, Sweden

**Keywords:** *S. aureus*, Staphylococcal infection, Indwelling device, Catheter infection, Infection severity score, Children with underlying diseases

## Abstract

**Background:**

Staphylococcus aureus (S. aureus) continues to be a significant pathogen responsible for a wide spectrum of infections with potentially fatal outcomes. The existing literature on S. aureus infection in children is still limited and highly heterogeneous, making it difficult to establish standardized indicators for complicated infections—especially in children with underlying medical conditions. Within this study we describe the clinical course and outcome of S. aureus infections in hospitalized pediatric patients with significant underlying disease or risk factors and assess whether clinical and/or laboratory features may be associated with the infectious course.

**Methods:**

Patients receiving inpatient care for S. aureus infection at the Karolinska University Hospital Huddinge, Sweden were identified by ICD-coding. Infection frequency, clinical course, underlying disease and medication were reviewed. Events were grouped according to severity (mild versus complicated) and subgroup analysis was performed.

**Results:**

Between 01/2004 and 12/2019, 38 patients with proven inpatient S. aureus infection were identified. These patients displayed a high number of recurrence accounting for a total number of 68 S. aureus infection episodes. All patients had significant underlying co-morbidities and risk factors. In 58 episodes (85.3%) an indwelling device was present. No S. aureus related mortality occurred and sequelae were extremely rare. Patients with complicated infection were more likely to display fever and elevated CRP at presentation, while leukocyte and neutrophil numbers did not significantly differ. Outpatient care prior to infection, local infection signs and a good or excellent status at presentation were associated with mild infection in our cohort.

**Conclusions:**

Recurrent infections were frequent among affected patients. Prompt antibiotic treatment upon suspicion of infection as well as the removal of indwelling devices may have contributed to the overall favorable outcomes in our cohort. Further studies are required to address the usefulness of identified variables to guide clinical decision making.

## Background

*S. aureus* is a highly versatile pathogen, which may be the source of invasive infections such as bacteremia, endocarditis, osteo-articular infections and, less common, pulmonary infections [1]. Moreover, *S. aureus* is the most common cause of skin and soft tissue infections (SSTIs) [2]. In children, *S. aureus* is a leading cause of both community- and healthcare-associated bacteremia. The estimated annual incidence of *S. aureus* bacteremia (SAB) ranges between 6.5 and 26 cases per 100,000 children [3–5]. Incidence and severity of staphylococcal infections are influenced by both host and bacterial risk factors [2, 6]. Certain methicillin-resistant *S. aureus* (MRSA) strains may increase the risk of complications [7, 8], albeit the overall effect on morbidity and mortality of pediatric patients in literature is variable [9–11]. This most likely reflects that the outcome is depending on local circumstances (e.g., respective patient cohort, access to health care, empiric management in high-risk groups) [12–14]. Despite advances in medical care, the mortality rate associated with invasive *S. aureus* infections in children, however, remains substantial, with reported rates from population-based cohort studies between 2 and 5% [4, 5], but higher rates in risk groups such as young infants with comorbidities [15–17]. Reported risk factors for SAB in children are young age (<1 year of age), presence of a central venous catheter (CVC) and socioeconomic factors, which may further influence the outcome of infection [3, 18, 19]. Healthcare associated infections in patients with chronic diseases are associated with a more complicated course and higher mortality [11]. In the absence of a CVC or heart diseases, immunosuppression and prematurity were found to be independent risk factors of pediatric SAB [16]. *S. aureus* is also a significant contributor to exit site, tunnel infections and dialysis-related peritonitis in children [20, 21]. While others have reported on catheter-associated infections in children with underlying diseases [22–24] or assessed risk stratification strategies in patients with enhanced infection vulnerability [25], data regarding the specific clinical course of *S. aureus* infections in pediatric patients with underlying diseases and multiple risk factors are still scarce. In the context of growing antibiotic resistance increasing the knowledge about staphylococcal infections in children with underlying diseases and risk factors remains crucial to guide clinical decisions. Consequently, this study was undertaken to characterize the spectrum of *S. aureus* infections, to assess recurrence, and to identify features associated with complicated versus mild course within a cohort of patients with significant underlying diseases.

## Methods

### Patients and definitions

**Fig. 1:**
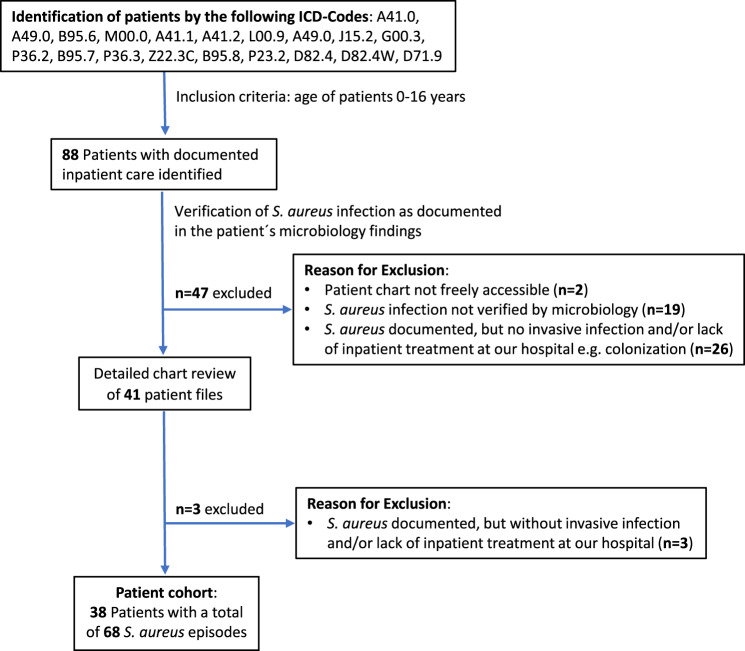
Cohort flow diagram

This retrospective, observational study was performed at the Pediatric Specialized Care Department, Karolinska University Hospital, Huddinge, encompassing the time period between 01/2004 and 12/2019 with the aim of assessing the clinical course and outcome of *S. aureus* infection in our patients with complex medical conditions. The hospital is a tertiary care hospital, predominantly admitting children with complex hematological, kidney and liver diseases. In addition, there is a NICU and a ward for general pediatrics. In contrast, there is no department for surgical, orthopedic or oncological diseases as these patients are treated at another site. Patients were identified by search of ICD-codes associated to presence of or predisposition for staphylococcus (Fig. 1). Only patients (0–16 years at study inclusion) with at least one documented *S. aureus* infection receiving inpatient care were included. Data regarding demographics and detailed course of documented *S. aureus* infections including comorbidities, treatment, presence of indwelling devices, duration of therapy, concomitant infections were extracted from the electronic patient records. Infectious episodes were categorized into mild and complicated. As no standardized classification for pediatric *S. aureus* infections currently exists, the scoring system was adapted from Munro et al. [26], complemented by our own clinical experience. The scoring system incorporated key clinical features associated to *S. aureus* infections (Table 1). Treatment failure was defined as persistent detection of *S. aureus* in blood or deep tissue on/after day 3 of antibiotic therapy. STROBE cohort reporting guidelines were used [27].

**Table 1:** Severity score definition

Criteria	Score Definition
**Localization of infection**	superficial-single = 0, superficial-multiple or invasive-contiguous = 1, invasive-multiple or non-contiguous = 2
**Treatment setting related to S. aureus**	outpatient or short term hospitalization (0–3 days)=0, medium length hospitalization (4–10 days) = 1, prolonged hospitalization (>10 days) = 2
**PICU admission**	no = 0/yes = 1
**Treatment failure**^**a**^	no = 0/yes = 1
**Sequelae**	no = 0/yes = 1
**Scoring: Mild infection = 0–1; Complicated infection ≥ 2**	

**Legend: PICU**: pediatric intensive care unit. ^a^**Treatment failure**: persistent detection of *S. aureus* in blood or

deep tissue on/after day 3 of antibiotic therapy

### Statistical analysis

Categorical variables are presented as frequencies and percentages, while continuous parametric variables are reported as means with standard deviation (SD) and range, and non-parametric variables as medians with interquartile range (IQR). Subgroup analysis: For parametric continuous variables Levene-test to assess equality of variances was used, followed by either two-tailed Student’s t-test (equal variance) or Welch test (non-equal variance). Differences between medians were assessed by non-parametric Median test, and Fisher´s exact test was employed for categorical variables. IBM SPSS Statistics v.29 was used (statistical significance: *p*-value ≤ 0.05).

## Results

### Study population

The study criteria with at least one documented inpatient *S. aureus* infection were met in 38 patients. The range of clinically or molecularly defined diagnoses was broad (Table 2). Six patients were deceased at the time of study inclusion. Yet, none of the deaths were related to *S. aureus*. Forty of the 68 *S. aureus* infections were derived from patients older than one year at the time of infection, 28 episodes originated from patients younger than one year (Table 2). All patients had an underlying disease at the time of *S. aureus* infection. When stratifying for the predominantly affected organ, kidney disease was present in 33 (48.5%) episodes. During 24 (72.7%) of these 33 episodes, patients received peritoneal dialysis (PD) and in 15 cases (45.5%) a CVC was present. The was no standardized approach regarding performance of an echocardiography. Among eight documented cases, seven were performed in patients with SAB and one in a patient with peritonitis due to hypotension. None was suggestive of endocarditis.

**Table 2:** Demographics and clinical characteristics of patient cohort

Demographics	Number	%	Median	IQR
Total number of patients	38			
Male	21	55.3		
Age in years of alive patients at study inclusion (*n* = 32)			7	10–4
Total numbers of *S. aureus* infections in the cohort	68			
Age of patients at *S. aureus* infection (all patients, **years**)	68		2	8–0
Age of patients at *S. aureus* infection (≥1 year in **years**)	40		6	9–2
Age of patients at *S. aureus* infection (<1 year in **months**)	28		3	6–2
			**Mean**	**SD (Range)**
Mean number of *S.aureus* infections per patient			1.8	1.3 (1–6)
**Underlying Disease Category (clinical/molecular diagnosis) (n = 38 patients)**^a^	**Number**	**%**		
Kidney disease	9	23.7		
Multisystem disorder	8	21.1		
Primary immunodeficiency	6	15.8		
Oncological disease	5	13.2		
Neonatal disease	4	10.5		
Hematological disease	2	5.3		
Infectious disease	2	5.3		
Liver disease	2	5.3		
**Most prominent organ dysfunction at the time point of S. aureus infection (n = 68 infection episodes)**^**b**^	**Number**	**%**		
Kidney disease	33	48.5		
Primary immunodeficiency	9	13.2		
Liver disease	8	11.8		
Hematological disease	6	8.8		
Oncological disease	6	8.8		
Prematurity	4	5.9		
Infectious disease	2	2.9		
**Specific features in patients with predominant kidney disease at the time point of S. aureus infection (n = 33)**^**c**^	**Number**	**%**		
PD	24/33	72.7		
Presence of CVC	15/33	45.5		
Kidney transplantion	11/33	33.3		

**Legend:**^a^addresses the primary underlying cause for medical care in the first place; ^**b**^based on expert opinion taking laboratory parameters and cause of medical care into account; ^c^might overlap; **CVC:** Central Venous Catheter; **IQR:** interquartile range (75^th^ Percentile − 25^th^ Percentile); **PD:** peritoneal dialysis: **SD**: standard deviation; Non-parametric data is displayed as whole numbers, parametric data with one decimal

### Infectious episodes

The most frequent invasive infections were SAB (*n* = 33, 48.5%) and peritonitis (*n* = 16, 23.5%) (Table 3). In 36 events (52.9%) there was a SSTI, mainly located at the insertion site of an indwelling device. *S. aureus* was recurrently detected in 41 episodes (60.3%) with detection at multiple sites in 50% of the events. Median time to positivity in SAB was 11 hours.

**Table 3:** Characteristics of infectious events

Overview of Events	Number	%	Treatment Characteristics in regards to S. aureus infection	Number	%
**Type of infection**^**a**^			Inpatient care	65	95.6
- Skin and soft tissue infection (SSTI)	36	52.9	Outpatient care	3	4.4
- *S. aueus* bacteremia (SAB)	33	48.5	Admission to PICU due to infection	4	5.9
- Peritonitis	16	23.5	Treatment failure^d^	9	13.2
- Ear infection (acute otitis media with tympanostomy tube otorrhea)	2	2.9	**Treatment Duration**	**Median**	**IQR**
- Urinary tract infection	2	2.9	Days of inpatient treatment, *n* = 61	9	15–6
- Pneumonia	2	2.9	Days of PICU treatment, *n* = 4	2	7–1
- Bone infection	1	1.5	Days of total antibiotic treatment, *n* = 63	14	21–10
**Patient Characteristics at Presentation**	**Number**	**%**	- Intravenous, *n* = 53	9	14–7
**General condition at presentation**^**b**^			- Oral, *n* = 36	7	18–6
- Excellent	8	11.8	- Intraperitoneal, *n* = 14	17	21–10
- Good	14	20.6	**Route of Antibiotic Therapy**	**Number**	**%**
- Stable	22	32.4	- Only intravenous	21	30.9
- Unwell	22	32.4	- Only oral	6	8.8
- Not known	2	2.9	- Only intraperitoneal	1	1.5
**Symptoms at presentation**			- Intravenous and oral	23	33.8
- Fever (documented Temperatur ≥ 38.5°), *n* = 59	28	41.2	- Intraperitoneal and oral	4	5.9
- Pain	18	26.5	- Intravenous and intraperitoneal	5	7.4
- Local infection signs	20	29.4	- Intravenous, intraperitoneal and oral	4	5.9
- altered peritoneal fluid	7	10.3	- other (unknown, no antibiotic therapy or only local antibiotic therapy)	4	5.9
	**Median**	**IQR**	**Indwelling Devices**^**a**^	**Number**	**%**
Days of symptoms prior to infection suspicion, *n* = 58	1	1–1	Any indwelling device present during event	58	85.3
**Clinical and Laboratory Findings at Presentation**	**Mean**	**SD (range)**	CVC present during any event	38	55.9
CRP (mg/L), *n* = 63	42.6	56.7 (0–309)	CVC present during SAB	27/33	81.8
Leukocytes (×10^9^/L), *n* = 58	11.3	8.0 (0.1–39.5)	PDC present during any event	24	35.3
Neutrophiles (×10^9^/L), *n* = 41	5.8	5.2 (0.0–22.2)	PDC present during peritonitis	16/16	100
Thrombocytes (×10^9^/L), *n* = 58	254	185 (6–810)	PEG present during any event	21	30.9
Temperature (°C), *n* = 59	38.3	1.0 (36.6–40.2)	Other devices (arterial line, ventilation tube,tracheostomy, SPC)	8	11.8
**Clinical and Laboratory Findings During Infection Episodes**	**Mean**	**SD (range)**	Removal of CVC (during any event)	26	38.2
Maximum CRP (mg/L), *n* = 63	110.9	102.4 (0–385)	Removal of PDC (during any event)	5	7.4
Peak temperature (°C), *n* = 50	38.9	1.0 (36.8–41.2)		**Median**	**IQR**
**Detection of S. aureus during events**	**Median**	**IQR/(range)**	Day of CVC removal; *n* = 26	3	8–2
Time to positivity in SAB (hours), *n* = 29	11	16–10/(3–34)	Day of PDC removal; *n* = 5	6	9–2
	**Number**	**%**	**Outcome of Infection**	**Number**	**%**
Detection of *S. aureus* at multiple sites during the same event	34	50.0	Restitutio ad integrum	67	98.5
Recurrent detection of *S. aureus* during the same event^c^	41	60.3	Sequelae^e^	1	1.5

**Legend:**^**a**^might overlap; ^b^as documented in the clinical assessment; ^c^includes also detection occurring up to 7 days prior to infectious event; ^d^defined as detection of *S. aureus* in blood or deep tissue on/after day 3 of antibiotic therapy. ^e^sequelae = defined as a medical consequence directly related to the *S. aureus* infection, in this case deep vein thrombosis during *S. aureus* cvc infection with development of post-thrombotic syndrome. **CVC:** central venous catheter; **CRP:** c-reactive protein; **IQR:** interquartile range (75^th^ Percentile − 25^th^ Percentile); **n:** number of included events for the respective data set; **PICU:** Pediatric Intensive Care Unit; **PDC**: peritoneal dialysis catheter; **S. aureus:***Staphylococcus aureus*; **SAB:***S. aureus* bacteremia. **SD**: standard deviation; **SPC**: suprapubic catheter; Non-parametric data is displayed as whole numbers, parametric data with one decimal

### Indwelling devices

In 85.3% of the episodes, an indwelling device was present (Table 3). A CVC was present in 38 (55.9%), a peritoneal dialysis catheter (PDC) in 24 (35.3%), and a percutaneous endoscopic gastrostomy (PEG) in 21 episodes (30.9%). Among 12 of these episodes both CVC and PDC were present. Five PDC and 26 CVC were removed during the infection episode. Detection of *S. aureus* on the CVC tip after removal was documented in 13 cases. In six of 33 SAB episodes there was no CVC present: in three of these there was a PEG, in two there was no indwelling device at all and in one there was a peripheral venous catheter, an arterial line and a tracheal tube.

### Antimicrobial prophylaxis and treatment

In 19 out of 68 (27.9%) infection episodes, patients received an antibiotic prophylaxis within 30 days prior to the event, which was ongoing at the time of presentation. Most of the patients were treated empirically with antibiotics at admission or upon clinical deterioration when infection was suspected during ongoing inpatient care of other cause. One patient did not receive any antibiotics, but the CVC was removed due to local infection signs. During 34 episodes, more than one different antibiotic was used concomitantly, and during 31 episodes intravenous and/or intraperitoneal treatment was combined or followed by an oral antibiotic course (Table 3).

### Immunosuppressive medication and transplantation

During 21 episodes, patients were on immunosuppressive medication and transplantation was documented in 20 episodes (Table 4). Median time between transplantation and infectious event was two months (IQR 13–0) for HSCT, 15 months (IQR 23–10) for liver transplantation (LTx) only and 46 months (IQR 52–18) for kidney transplantation (KTx) only. All patients with HSCT still had a CVC at the time of infection. The respective liver and/or kidney transplanted patients still had significant underlying diseases: Some of them displayed varying degrees of graft failure or suffered from nutritional problems. In 2/7 episodes in kidney transplanted patients there was still a CVC present. Four episodes originated from one patient who underwent both LTx and KTx but was again on PD due to kidney graft failure.

**Table 4:** Risk factors

Risk factors at time point of S. aureus infection	Number	%
**Transplantation for underlying disease (n = 68)**	20	29.4
Hematopoietic stem cell transplantation	5	7.4
Kidney transplantation only	7	10.3
Liver transplantation only	4	5.9
Kidney and liver transplantation	4	5.9
**Immunosuppression and related therapies (n = 68)**^**a**^		
Ongoing chemotherapy	9	13.2
Ongoing immunosuppressive medication	21	30.9
**Hospital related factors (n = 68)**		
Ongoing inpatient care	22	32.4
Operation for underlying disease	3	4.4
**Laboratory values**		
Neutropenia (<1000 cells/µl), *n* = 59	8	13.6

^a^might overlap, **n:** number of included events for the respective data set

### Microbiology and resistance pattern

Susceptibility testing was available for 32/33 isolated strains from blood, for all 16 strains from peritoneal fluid and for 43 strains from other infection sites (e.g., tissue, skin, urine). Among them, two strains from blood and one from tissue were MRSA (e.g., resistant to isoxazolyl penicillin). Other resistances found (including the MRSA isolates) were resistance against fusidic acid (*n* = 3), clindamycin (*n* = 3), trimethoprim/sulfamethoxazole (*n* = 2), gentamicin (*n* = 1). During 18 *S. aureus* infections (26.5%) a co-infection was detected.

### Initial presentation and hospital course

During 46 episodes (67.6%), the patients were outpatient prior to the *S. aureus* infection while during 22 episodes (32.4%) they developed the *S. aureus* infection during an inpatient hospitalization period, with cultures obtained more than 48 hours after admission (Table 3). Median time from symptom onset to suspicion of an infection and treatment start was 1 day, based on day-level assessment. Most frequent symptoms at presentation were fever ≥ 38.5 °C (41.2%), (abdominal) pain (26.5%), local infection signs (e.g., redness, tenderness, local swelling) (29.4%) or altered peritoneal fluid (10.3%). The general status at presentation as documented by the attending clinician ranged between excellent, i.e. not displaying any symptoms at all and unwell, indicating that the patient appeared clinically ill. In 95.6% of the events, the patients received inpatient care.

### Persistence or recurrence of infection

Primary treatment failure was documented in nine episodes (13.2%). In all of these episodes one or more indwelling devices were present and none was removed before day 3. None of the strains in treatment failure was resistant. 16 patients (42.1%) experienced more than one episode, with 10 patients showing two infections and two patients each having three, four and six infections. Ten events (14.7%) were estimated to be related to a previous due to recurrence of infection after antibiotic therapy within 1 month. These episodes derived from six patients. In all these 10 events an indwelling device was present (CVC *n* = 2, PDC *n* = 3, CVC and PDC *n* = 5). None of the patients had a device removed during the initial event, all patient ultimately underwent removal of the respective infected device. This prevented additional early relapse in 5 out of 6 patients.

### Subgroup analysis

Based on the applied scoring system, 17 infections were classified as mild and 49 infections as complicated (Table 5). Two infectious events could not be scored due to hospital transfer. During complicated *S. aureus* infection, patients were more likely to have an indwelling device (93.9% vs. 58.8%; *p* = 0.002), while there was no significant difference in the rate (46.9% vs. 35.3%; *p* = 0.572) and time point of device removal (median day 6 vs. day 2; *p* = 0.169). A mild infection was associated to prior outpatient care (100% vs. 57.1%; *p* < 0.001), local infection signs (70.6% vs. 14.3%; *p* < 0.001) and a good or excellent general status at presentation (88.2% vs. 12.5%; *p* < 0.001), while fever (≥38.5°C) at presentation was more common among complicated events (59.1% vs. 7.1%; *p* < 0.001). Regarding laboratory values, the initial CRP level was higher (mean 51.5 mg/L vs. 14.5 mg/L; *p* < 0.001) and the thrombocyte counts were lower (mean 216 × 10^9^/L vs. 352 × 10^9^/L; *p* = 0.003) during complicated versus mild infectious events, while leukocyte (mean 11.5 × 10^9^/L vs. 9.6 × 10^9^/L; *p* = 0.284) or neutrophil counts (mean 6.3 × 10^9^/L vs. 4.0 × 10^9^/L; *p* = 0.078) were not significantly different. During the infection course, both peak temperature (mean 39.2 °C vs. 37.6 °C; *p* < 0.001) and maximal CRP levels (mean 128.6 mg/L vs. 52.1 mg/L; *p* = 0.013) were higher during complicated infections.

**Table 5:** Comparison between mild versus complicated *Staphylococcus aureus* infections

	Mild (n = 17/66; 25.8%)		Complicated (n = 49/66; 74.2%)		T	p	Cohen’s d
Demographics	Median	IQR	Median	IQR			
Age in years at *S. aureus* infection	5 (*n* = 17)	9–1	2 (*n* = 49)	8–0		0.573	
	**Number**	**%**	**Number**	**%**			
Male Gender	12	70.6	27	55.1		0.391	
**Risk factors**	**Number**	**%**	**Number**	**%**			
Any indwelling device	10	58.8	46	93.9		**0.002**	
Presence of CVC	6	35.3	30	61.2		0.091	
Presence of PDC	3	17.6	20	40.8		0.139	
Removal of indwelling device: yes	6	35.3	23	46.9		0.572	
	**Median**	**IQR**	**Median**	**IQR**			
Time point of removal of indwelling device (days)	2 (*n* = 6)	4–2	6 (*n* = 23)	10–2		0.169	
**Clinical aspects**	**Median**	**IQR**	**Median**	**IQR**			
Days of symptoms prior to infection suspicion	1 (*n* = 11)	1–1	1 (*n* = 46)	1–1		0.581	
	**Mean**	**SD (range)**	**Mean**	**SD (range)**			
Temperature at presentation (°C)	37.4 (*n* = 14)	1 (36.6–40.2)	38.6 (*n* = 44)	0.9 (36.6–40)	−4.564	**<0.001**	−1.400
	**Number**	**%**	**Number**	**%**			
Outpatient care prior to *S. aureus* infection	17 (*n* = 17)	100	28 (*n* = 49)	57.1		**<0.001**	
Fever at presentation (temp ≥ 38.5°C)	1 (*n* = 14)	7.1	26 (*n* = 44)	59.1		**<0.001**	
Local infection signs at presentation	12 (*n* = 17)	70.6	7 (*n* = 49)	14.3		**<0.001**	
Good/excellent general status at presentation^a^	15 (*n* = 17)	88.2	6 (*n* = 48)	12.5		**<0.001**	
**Laboratory findings**	**Mean**	**SD (range)**	**Mean**	**SD (range)**			
** At presentation**							
Leukocyte numbers (×10^9^/L)	9.6 (*n* = 14)	4.5 (1.8–19.4)	11.5 (*n* = 43)	8.6 (0.1–39.5)	−1.084	0.284	−0.246
Neutrophil numbers (×10^9^/L)	4.0 (*n* = 9)	2.1 (0.4–6.1)	6.3 (*n* = 32)	5.7 (0–22.2)	−1.818	0.078	−0.435
CRP (mg/L)	14.5 (*n* = 14)	18.2 (0–54)	51.5 (*n* = 48)	61.7 (0–309)	−3.639	**<0.001**	−0.668
Thrombocyte numbers (×10^9^/L)	352 (*n* = 15)	122 (21–492)	216 (*n* = 42)	193 (6–810)	3.125	**0.003**	0.763
** During infection**							
Peak CRP level (mg/L)	52.1 (*n* = 14)	68.7 (0–189)	128.6 (*n* = 48)	105.7 (0–385)	−2.548	**0.013**	−0.774
Peak temperature (°C)	37.6 (*n* = 9)	0.9 (36.8–39.5)	39.2 (*n* = 40)	0.8 (37.5–41.2)	−4.851	**<0.001**	−1.790
**Infection characteristics**	**Number**	**%**	**Number**	**%**			
Skin and soft tissue infection	13 (*n* = 17)	76.5	21 (*n* = 49)	42.9		**0.024**	

**Legend:**^a^as documented in the clinical assessment. **CVC:** central venous catheter; **CRP:** c-reactive protein; **n:** number of available events for the respective data set; **IQR:** interquartile range (75^th^Percentile − 25^th^Percentile)

**PDC**: peritoneal dialysis catheter; **S. aureus:***Staphylococcus aureus*; **SD**: standard deviation. **Statistics:** differences between medians were assessed by non-parametric Median test, categorial variables by Fisher´s exact

test and parametric continuous data by two-tailed Student´s t-test (if equal variance) or Welch test (if non-equal variance). Missing values handling by pairwise exclusion. Non-parametric data is displayed as whole

numbers, parametric data with one decimal. Significance threshold *p*-value ≤ 0.05

## Discussion

When a pediatric patient with multiple co-morbidities, several indwelling devices and on immunosuppressive therapy presents with signs of exit site or systemic infection, it may be challenging to estimate at first sight whether clinical symptoms are related to SAB and whether the clinical course will be mild or complicated. Within this study, we therefore assessed the spectrum, recurrence and clinical courses of *S. aureus* infections in a patient cohort with significant underlying diseases and found that fever, lack of good/excellent general status and higher CRP levels at presentation were associated with a complicated course in this high-risk population.

The patients in our cohort had a wide range of underlying chronic diseases and displayed multiple known risk factors associated with *S. aureus* infections. As they belonged to a vulnerable group, they were promptly treated with empiric broad-spectrum antibiotics upon suspicion of infection. Still, there was a high rate of complicated infections and we found a marked recurrence rate in individual patients.

There is currently no evidence-based consensus on the definition of a complicated pediatric *S. aureus* infection [3]. Given the lack of high-quality pediatric clinical trials, evidence assessment is frequently derived from adult trials and recommendations are predominantly expert-based. Within their consensus paper, McMullen et al. suggested features such as persistent bacteremia and/or fever beyond 72 hours of targeted therapy, multifocal sites of infection, endocarditis and complex local diseases involving multiple adjacent tissues as potential signs of complicated SAB [3]. Definitions used by others include SAB with additional presence of major organ involvement [10]. When defining a complex SAB by the extend of *S. aureus* infection localization and the severity of the clinical course, Munro et al. have shown that a complex infection is associated with prolonged hospitalization [26]. Expanding on these previous suggestions we were able to categorize the infectious events in mild and complicated, which aligned well with the clinical assessment.

Epidemiological, population-based studies suggest that bone and joint infections are most frequent in the pediatric population [5]. In contrast to other studies reporting predominantly on patients with SAB and SSTI or bone/joint focus [10, 26, 28], there was only one patient who displayed a bone infection in our cohort, while SSTIs were evident in about half of the events. Interestingly, the presence of a SSTI as well as local infection signs at presentation were associated with a milder infection in our cohort. Whether this reflects an early and efficient treatment of localized infection or potentially stronger compromised immune defense mechanisms in patients with absent local infection signs leading directly to a more severe course, remains to be deciphered.

Elevated CRP has been reported to be associated with a worse outcome in children with SAB [26, 29]. In our cohort, CRP levels were significantly lower both at admission and throughout the clinical course in mild compared to complicated infections. Munro et al. suggested that a CRP-value < 75 mg/L at 48 hours may potentially aid in clinical decision making towards a step-down to oral antibiotics [26]. In our cohort, mean peak CRP levels did not exceed 53 mg/L in mild infection. However, the highest reported peak value during mild infections was 189 mg/L. In addition, a few patients did not show marked CRP elevation in spite of being classified as complicated infection. Thus, even though we can confirm CRP-levels as a valuable indicator for the clinical course in our cohort as well, its use alone appears to be insufficient to correctly classify all infectious episodes in patients with underlying diseases. In this context, it needs to be considered that the inflammatory response to *S. aureus* may be masked in the setting of immunosuppressive treatments or underlying immunodeficiencies [6]. Other factors, which were found to be associated to a milder infection in our cohort were outpatient care prior to infection, temperature < 38.5° and a good or excellent general status at presentation. Within our study cohort, there were no significant differences in leukocyte or neutrophil counts between mild and complicated course. It is, however, important to underline that several patients displayed neutropenia and where thus treated rapidly upon presentation of fever/infection signs. Although neutropenia is a well-known risk factor for staphylococcal infection, it was not associated with a more complicated course within our high-risk cohort, most likely reflecting adherence with national fever in neutropenia guidelines.

Within our cohort, the number of patients with any indwelling device was clearly enriched in complicated events. The type of indwelling device itself did, however, not reach significance, most likely due to the high rate of various devices within the whole cohort. From a clinical perspective, another challenging question is whether an indwelling device should be removed. Given the risk of biofilm formation by *S. aureus*, prompt catheter removal has been advocated in treatment guidelines for central line associated SAB unless there are significant contraindications for line removal [30]. In this context, CVC salvage may be a valuable option for uncomplicated cases as demonstrated in a study in which CVC salvage was successful in 44/56 attempts [31]. Predictors of salvage failure identified across different studies were the presence of an implantable venous access device, history of catheter infection, initial presence of severe sepsis and polymicrobial infection, external signs of catheter infection, MRSA as well as inadequate empiric treatment [22, 32, 33]. Reported treatment failure and recurrence rates in children vary depending on the respective cohorts and definitions [9, 18, 34]. In our cohort, primary treatment failure was considerable, occurring in 13.2% of events, and there was a high early relapse rate of 14.7% within 1 month. While we did not observe any significant difference between the removal of device in the subgroup analysis in regards to the overall clinical course, it became evident that when evaluating the 6 patients with relapses, device removal was required to prevent further early relapses. This, in line with data from other studies [23, 35], suggests that eradication of *S. aureus* in high-risk children may be difficult without device removal.

Reported mortality rates in children due to *S. aureus* are variable. A large epidemiological study in 57,794 children reports on an overall death rate of 2% [36]. In contrast, a high rate of 15% was found in the study by Araya et al. where frequent ICU admission for pneumonia and multifocal disease was present [17]. We hypothesize that the overall favorable outcome in our high-risk cohort may be attributed to rapid empiric treatment initiation upon clinical suspicion of infection. Of note, considerably longer duration of symptoms were documented in a study comparing outcome of primary admission to rural versus urban sites (mean of 4.1 vs 3.0 days, respectively), likely contributing to prolonged bacteremia and worse outcome in patients from rural areas [18]. Given the enhanced risk for complications in persistent SAB [9, 35], our study underlines the benefit of rapid treatment initiation in patients with severe underlying conditions.

### Limitations of the study

This study characterizes the risk factors and infection burden in a selective patient cohort, i.e., predominantly patients with significant underlying disease and co-morbidities presenting to the hematological, hepatological and kidney disease department, thus limiting generalization to other patient groups. The retrospective nature of the study, identification via ICD coding and exclusion of patients with inaccessible patient files may have led to a selection bias. The absolute number of events is small and the heterogenous patient composition with several risk factors present in individual patients limits a more in-depth statistical analysis. Still, the study provides some important findings in this patient cohort as the respective episodes were reviewed in detail and recurrent episodes over the study time period from the same individual were taken into account. Region Stockholm has a long-standing history of using digital journals. This allows both a comprehensive and detailed analysis of associated factors and thorough assessment of the respective clinical course notwithstanding the limitations of retrospective data collection. Clearly, a prospective study covering all identified areas of interest, ideally using microbiological identification of *S. aureus* and not only ICD coding as the starting point for patient selection, is required for further validation.

## Conclusions

In pediatric patients with underlying diseases and co-morbidities the infectious burden of *S. aureus* may be significant and recurrence may be common. Rapid empiric treatment, low rates of antibiotic resistance but ultimately also removal of indwelling devices likely contributed to the overall favorable outcome of the infectious episodes in our high-risk cohort. Further studies are needed to address the validity of identified variables in predicting a complicated course in both patients with underlying diseases but also in otherwise healthy individuals with *S. aureus* infections.

## Data Availability

The datasets generated and/or analysed during the current study are not publicly available due to patient confidentiality but are available from the corresponding author on reasonable request.

## Abbreviations

CRP: C-reactive protein
CVC: Central venous catheter
HSCT: Hematopoietic stem cell transplantation
ICD: International Classification of Diseases
IQR: Interquartile range (=75^th^ percentile − 25^th^percentile)
MRSA: Methicillin resistant *S. aureus*
NICU: Neonatal intensive care units
PD: Peritoneal dialysis
PDC: Peritoneal dialysis catheters
PEG: Percutaneous endoscopic gastrostomy
SAB: *Staphylococcus aureus* bacteremia
*S. aureus*: *Staphylococcus aureus*
SSTI: Skin and soft tissue infection
